# Endovascular Treatment of Large Core Infarcts: no Limits?

**DOI:** 10.1007/s00062-024-01472-6

**Published:** 2024-11-14

**Authors:** Martin Bendszus

**Affiliations:** Dept. of Neuroradiology, University of Heidelberg, 69120 Heidelberg, Germany

In September 2024, the TESLA trial has finally been published as the last of six studies on the endovascular treatment of large core infarcts [1–6]. Despite the overarching clinical research question, all of these trials have distinct features regarding patient populations, inclusion criteria and endpoints. Overall, there is a clear benefit in terms of a better clinical outcome in patients receiving endovascular thrombectomy, no matter if selected on basis of MRI, plain CT or perfusion imaging. This applies only in patients with a good premorbid modified Rankin Scale (mRS) of 0–1 [1–3, 5, 6] and 0–2 [4], respectively. In terms of mortality, there was a significant reduction in two trials [4, 5]. Overall, the rates of poor clinical outcomes (mRS 4–5) were reduced in all trials. These favorite clinical outcomes persisted up to 12 months after treatment [7, 8]. Interestingly, in subgroup analyses the ASPECTS prior to therapy was not a strong prognostic marker. Only one trial (TESLA) failed to reach the prespecified endpoint [6]. Despite an overall increase in moderate clinical outcomes (mRS 0–3) from 20% in best medical treatment to 30% in the endovascular group, the primary endpoint to show superiority of endovascular treatment with a 97.5% probability was not achieved. TESLA allowed inclusion of patients up to 24 h after stroke onset with an ASPECT score of 2–5. While the ASPECT score was not a determinant for outcome, patients treated after 6 h showed no clinical benefit after endovascular thrombectomy. This time dependency of treatment effects was not present in other trials [2–4]. The upcoming meta-analysis on individual patient data will hopefully reveal prognostic factors prior to treatment in this particular patient population with a moderate prognostic profile.
